# Hypolipidemic and anti‐atherogenic activities of crude polysaccharides from abalone viscera

**DOI:** 10.1002/fsn3.1548

**Published:** 2020-04-20

**Authors:** Binxiong Liu, Zhen Jia, Changcheng Li, Jinquan Chen, Ting Fang

**Affiliations:** ^1^ College of Food Science Fujian Agriculture and Forestry University Fuzhou China; ^2^ Engineering Research Center of Ministry of Education: Fujian‐Taiwan Featured Marine Food Processing and Nutritional Health Fuzhou China

**Keywords:** abalone viscera polysaccharides, anti‐atherosclerosis, hypolipidemic, infrared spectroscopy

## Abstract

This study was performed to evaluate the hypolipidemic and anti‐atherogenic activities of the crude polysaccharides extracted from abalone viscera (AVCP). The major functional groups of purified polysaccharides were analyzed by infrared spectroscopy (IR). Male Kunming mice (SPF) were divided into six groups and were treated with normal diet or high‐fat diet with AVCP or Xuezhikang (hypotensive drug) for 5 weeks. Physicochemnical analysis of AVCP showed the presence of 60.4% polysaccharides, 17.9% protein, 6.0% fat and 10.9% moisture. The IR analysis of AVP showed the presence of functional groups of sugar moiety and sulfate groups. The results demonstrated that AVCP not only led to significant reduction of total cholesterol (TC), triglycerides (TG), low‐density lipoprotein cholesterol (LDL‐C), and increase of high‐density lipoprotein cholesterol (HDL‐C) in plasma, but also to significant increments of malondialdehyde (MDA) and superoxide dismutase (SOD) activities. However, AVCP played no role in mice weight. Furthermore, the results of the photomicrograph of liver tissue showed that AVCP reduced lipid droplets and prevented the disordered structure of the liver. The results suggested that AVCP exhibited significantly hypolipidemic and anti‐atherogenic activities.

## Graphical abstract

The antihyperlipidemic and antiatherosclerotic activities of crude polysaccharide from abalone viscera were investigated by rat experiment. The results showed it not only significantly reduce plasma total cholesterol (TC), triglyceride (TG), low‐density lipoprotein cholesterol (ldl‐c), and high‐density lipoprotein cholesterol (hdl‐c), but also significantly improve the activity of malondialdehyde (MDA) and superoxide dismutase (SOD).

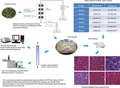

## 1 INTRODUCTION

Abalone (*Haliotis discus hannai*) is one of the most valuable marine mollusks throughout the world. In general, the by‐products from the abalone processing, viscera, may account for 15%–25% of the total body weight of raw material (Zhou et al., 2012). This visceral matter is considered as an inedible part and normally discarded as industrial waste, which may pose a disposal problem due to the ease of spoilage and environmental pollution (Zhu et al., 2008). As reported, abalone viscera contained many health‐beneficial substances such as polysaccharides, oil, peptides, and hormones (Su, Liu, Wang, & Wu, 2010), indicating abalone viscera has a potential to be reclaimed. Recently, abalone viscera polysaccharides (AVCP) have been gained more attention for their various biological effects, such as antioxidant (Liu, Zhu, Sun, & Liu, 2011), antifatigue (Guo et al., 2011), and immunostimulatory activities (Wang et al., 2015).

Atherosclerosis, the complex interaction of serum cholesterol with the cellular components of the arterial wall, is the main cause of cardiovascular diseases and results in a high incidence of death continuously (Pang et al., 2010). The concentrations of total cholesterol (TC), triglycerides (TG), low‐density lipoprotein cholesterol (LDL‐C), and high‐density lipoprotein cholesterol (HDL‐C) in blood are the major risk factors in the pathogenesis of atherosclerosis (Morishita, Iwahashi, & Kido, 1986; Wu, 2007). Moreover, lipid peroxidation is also associated with the development of atherosclerosis (Chanet et al., 2012). Nowadays, atorvastatin drugs are widely used to treat atherosclerosis since it can decrease the level of plasma LDL‐C and has a strong antioxidant effect. Unfortunately, these drugs have adverse effects, such as a headache, abdominal distension, and insomnia (Lusis, 2000), which are harmful to the human body. Therefore, alternative methods are needed to against atherosclerosis.

It has been indicated that natural polysaccharides extracted from plants and microorganisms have hypolipidemic activity in general and can be developed as novel potential hypolipidemic agents (Lankin et al., 2003; Lee & Park, 2013). The polysaccharides of *Lonicera japonica* have effect on lowering blood lipids in vivo and scavenging superoxide and hydroxyl radicals in vitro (Chen et al., 2011; Li, Zhang, & Ma, 2010). However, as far as our literature survey could ascertain, hypolipidemic and anti‐atherogenic activities of AVCP have not been studied.

Therefore, the objective of the study was to investigate the effect of AVCP on the hypolipidemic and anti‐atherogenic activities.

## 2 MATERIALS AND METHODS

### 2.1 Materials and equipment

Frozen abalone (*Haliotis discus hannai*）viscera, stored at −18°C, were kindly provided by Zhangzhou Ousheng Food Co., Ltd. The frozen abalone viscera was thawed at room temperature prior to experiment.

**FIGURE 1:**
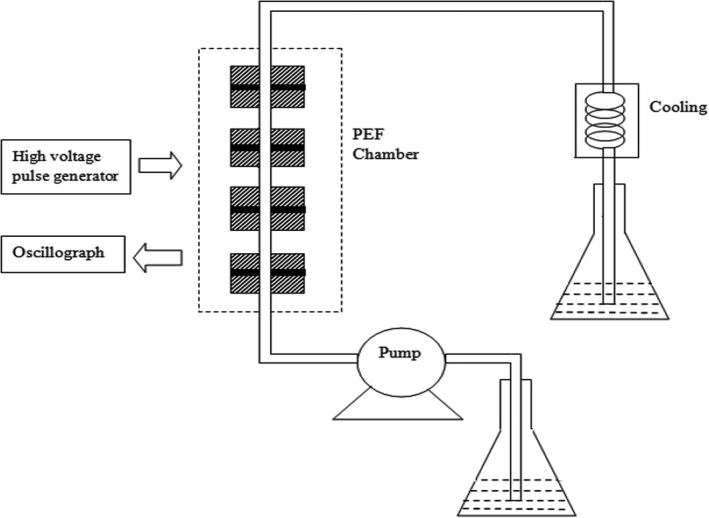
Schematic of high‐intensity pulsed electric fields processing apparatus

**FIGURE 2:**
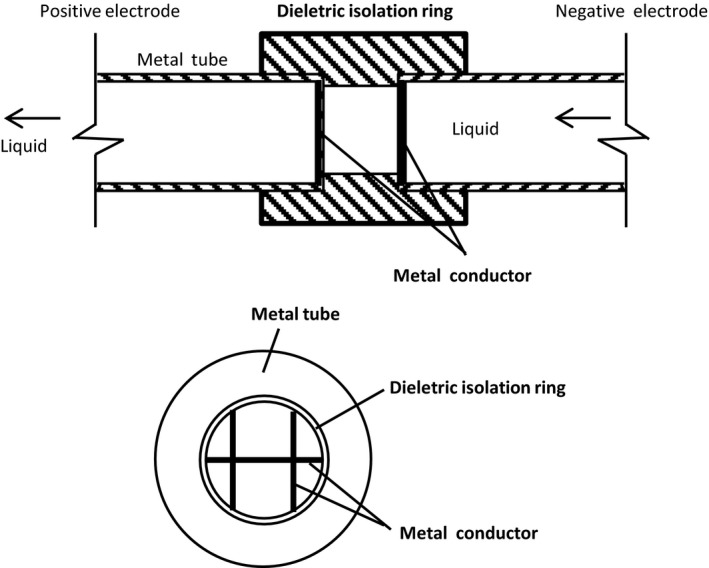
Structure diagram of pulsed electric field treatment chamber

Pulsed electric field (PEF) system (Figure 1) was self‐designed by Professor Jinquan Chen in Fujian Agriculture and Forestry University, China. It consists of a high‐voltage pulse generator, an oscillograph, a coaxial liquid material treatment chamber, a pump, and an ice‐water bath. The structure diagram of the PEF treatment chamber is shown in Figure 2. There was a pair of parallel electrode bars, locating at two ends of the treatment chamber. The positive electrode received high‐voltage pulse power and was grounded at the negative electrode. Pulse shape (square shape bipolar) was monitored online with an oscilloscope during treatment. The diameter of the treatment zone was 6 mm.

### 2.2 Chemicals, experimental diets and drugs

#### 2.2.1 Chemicals

Neutral protease (Neu) was obtained from Beijing Donghua Qiangsheng Biotechnology Co. Ltd. Sulfuric acid was obtained from Beijing Chemical Works. Sodium hydroxide, D‐glucose anhydrous, alcohol, phenol, 3, 5‐dinitrosalicylic acid, and sodium hydrogen sulfite were obtained from Sinopharm Chemical Reagent Co., Ltd. All the chemicals were of analytical grade. Triglyceride kits, total cholesterol reagent kits, high‐density lipoprotein cholesterol kits, low‐density lipoprotein cholesterol kits, superoxide dismutase kit, and malondialdehyde test kit were obtained from Nanjing Jiancheng Biology Engineering Institute.

#### 2.2.2 Experimental animals

Specific pathogen‐free (SPF) male Kunming mice (15 ± 2 g) were obtained from the Wu Laboratory Animal Center. Mice were acclimatized for 1 week before the commencement of experiments under standard environmental conditions of temperature at 25 ± 2°C and 50%–60% of relative air humidity and 12 hr dark/light cycle with free access to normal diet and water. The animal facilities and all experimental procedures were carried out according to the National Institutes of Health (NIH) guidelines.

#### 2.2.3 Experimental diets and drugs

Normal diet was purchased from the local market, the nutritional ingredients of which were in accordance with nutritional standards. The high‐fat feeding was self‐made which contained 80.8% normal feeding, 5% lard, 4% cholesterol, 0.2% bile salt, and 10% protein powder. Xuezhikang capsule (an approved traditional Chinese medicine for the treatment of hyperlipidemia bought from pharmacy) was obtained from Beijing Beida Weixin Biological Technology Co., Ltd.

### 2.3 AVCP extraction

One‐kilogram abalone viscera mixed with 2 kg of water was pulped, removing impurities and connective tissues. The abalone viscera solution was filtered, homogenized, and centrifuged successively. One liter of supernatant solution was treated by PEF with a flow velocity of 100 ml/min, and the electric field intensity and pulse number were 30 kV/cm and 12, respectively. After processing for 10 min, the solution was hydrolyzed by the neutral protease (2.4 × 10^4^ U/g) for 2.5 hr at 50°C. The enzyme was inactivated by increasing the temperature to 100°C and kept for 5 min. Afterward, the hydrolyzate was centrifuged at 2,670 *g* for 15 min. The supernatant was precipitated via adding 95% alcohol with the ratio of 1:3 (v/v) and stored at 4°C overnight. The precipitates were centrifuged at 2,670 *g* for 15 min to obtain the sediment, which was then dried by vacuum freeze dryer to yield the abalone viscera crude polysaccharides (AVCP) powder.

### 2.4 AVCP purification

Abalone viscera crude polysaccharide (1 mg/ml) was mixed with Sevag solvent (chloroform:n‐butanol = 4:1 (v/v)) at ratio of 3:1. The mixture was thoroughly stirred with a magnetic stirrer for 30 min and then centrifuged at 2,809 *g* for 10 min. The solution was separated into three layers. The upper layer was harvested and treated again as mentioned above until the middle layer with protein precipitates disappeared. The supernatant was dialyzed using MD34‐3500 membrane (Beijing Solarbio Science & Technology Co., Ltd) against running tap water for 48 hr and distilled water for another 24 hr at ambient temperature, changing water per 2 hr. The dialysate was dried by the vacuum freeze dryer to obtain the preliminary purified polysaccharides powder.

One hundred milligrams of the preliminary purified polysaccharides was dissolved in distilled water. The mixture was centrifuged to discard the sediment. The supernatant was loaded into a DEAE‐52 chromatography column (Beijing Solarbio Science & Technology Co., Ltd), eluted with distilled water, 0.1, 0.3, and 0.6 mol/L of NaCl solution in sequence at flow rate of 0.5 ml/min. The solution out of column was collected in each tube per 6 min. The content of polysaccharides in each tube was determined by the phenol–sulfuric acid method (Wang, Wang, & Pang, 2007). The collected samples were combined, concentrated, mixed with 95% alcohol, and centrifuged. The precipitate was dissolved in distilled water and then dried to obtained the purified polysaccharides powder (AVP).

### 2.5 Physicochemnical analysis of AVCP

#### 2.5.1 The content of crude polysaccharides in AVCP

Total sugar content and the reducing sugar content of AVCP were individually measured using the phenol–sulfuric acid method and 3,5‐dinitrosalicylic acid method (DNS; Zhao, Xue, & Li, 2008). The percentage of crude polysaccharides in AVCP was calculated using Equation 1:

(1) $$C\left(\%\right)=\frac{C_{t}-C_{r}}{C_{t}}\times 100\%$$

where *C*_t_ and *C*_r_ represent the total sugar content (%) and reducing sugar content (%), respectively.

#### 2.5.2 The content of protein in AVCP

Protein in AVCP powder was analyzed with an Automatic Kjeldahl Analyzer (Shanghai HongJi Instrument Co., Ltd). One hundred fifty milligram of AVCP powder was placed into a 500‐ml Kjeldahl nitrogen digestive tube and mixed with 0.2 g of anhydrous cupric sulfate, 6.0 g of potassium sulfate, and 10 ml concentrated sulfuric acid. Another digestive tube without AVCP but with other chemical agents, as control, was prepared. The tubes were transferred into the digestive furnace and digested at 250°C until the clear blue‐green liquid appeared. After cooling to ambient temperature, the content of protein was determined according to national standards (SAC, 2016). All assays were performed triplicate.

#### 2.5.3 The content of fat and water in AVCP

The contents of fat and water in AVCP were determined according to GB5009.6‐2016 (2016); GB5009.3‐2016 (2016).

### 2.6 Gas chromatography analysis of AVP

Ten milligrams of each standard monosaccharide (L‐rhamnose, D‐xylose, D‐mannose, D‐glucose, D‐galactose, fructose, glucuronic acid, fucose) was mixed with 10 mg of hydroxylamine hydrochloride and dissolved in 0.5 ml of pyridine. The solution was kept in water bath at 90°C for 30 min. After cooling down, the solution was added with 0.5 ml of anhydrous acetic anhydride and reacted in water bath at 90°C for 30 min. The reaction product was analyzed by gas chromatography (GC; Bai & Zhang, 2011).

### 2.7 Infared spectroscopy analysis of AVP

The purified abalone viscera polysaccharide was mixed with KBr powder at ratio of 1:100 (g/g) and pressed into a pellet. The pellet was analyzed by infrared spectroscopy (IR) within the frequency range of 4,000–400 cm^−1^ (Kumar, Joo, Choi, Koo, & Chang, 2004).

### 2.8 Determination of hypolipidemic and anti‐atherogenic activities

#### 2.8.1 Animal grouping and treatment schedule

A total of 60 mice were weighed and randomly divided into six groups of 10 mice: blank control group (A), positive control group (B), negative control group (C), low‐dose group (D), middle‐dose group (E), and high‐dose group (F), and fed as shown in Table 1. All mice had free access to potable drinking water, and each mouse was weighed once a week during the experiment. After 5 successive weeks for administration, eyeball blood was collected in heparin (anticoagulant) tubes from each mouse after gavage for 1 hr and centrifuged immediately at 500 *g* for 10 min. The supernatant serum was obtained for biochemical analysis. The animals were thereafter killed by cervical dislocation. The livers were harvested and perfused with normal saline (0.9%) at 4°C. After then, the hepatic tissues were sectioned (5 mm × 5 mm × 2 mm) and immersed in a 10% formaldehyde solution for histopathological assessment.

**TABLE 1:** The grouping and feeding of mice in this experiment

Groups	Diet	Gavage	Concentration	Dose
Group A	Normal diet	Saline	0.9%	1.2 ml
Group B	High‐fat diet	Xuezhikang capsule	400 mg/kg bw.d	
Group C		Saline	0.9%	
Group D		AVCP	200 mg/kg bw.d	
Group E		AVCP	400 mg/kg bw.d	
Group F		AVCP	600 mg/kg bw.d	

#### 2.8.2 Determination of lipid and lipid peroxidation index

The levels of malondialdehyde (MDA) and superoxide dismutase (SOD) activities in serum were individually determined using Malondialdehyde kit and Superoxide dismutase kit. The levels of TG, TC, LDL‐C, and HDL‐C were separately analyzed using triglyceride kit, total cholesterol reagent kit, low‐density lipoprotein cholesterol kit, and high‐density lipoprotein cholesterol kit.

#### 2.8.3 Histopathological assessment

Hepatic tissues were embedded in paraffin and stained with hematoxylin and eosin, and then examined by an electronic microscope.

### 2.9 Statistical analysis

Statistical analysis was performed using SPSS, Version 13.0 (SPSS, Inc.). All the results were presented as mean ± standard deviation (*SD*) for ten mice in each group. *p* < .05 means significant (data were subjected to the analysis of variance [ANOVA], followed by mean comparisons by Duncan's multiple range test at *p* < .05).

## 3 RESULTS

### 3.1 Physicochemical analysis of AVCP

As shown in Table 2, the extraction rate of AVCP was 3.0%, the mean values of crude polysaccharides, protein, fat, and water in AVCP powder were 60.39 ± 0.23%, 17.88 ± 1.39%, 6.02 ± 0.09%, and 10.94 ± 0.21%, respectively.

**TABLE 2:** Analysis of abalone viscera crude polysaccharides

Ingredients	Average content (%)
Crude polysaccharides	60.39 ± 0.23
Protein	17.88 ± 1.39
Fat	6.02 ± 0.09
Water	10.94 ± 0.21

### 3.2 Purification of AVCP and GC analysis

**FIGURE 3:**
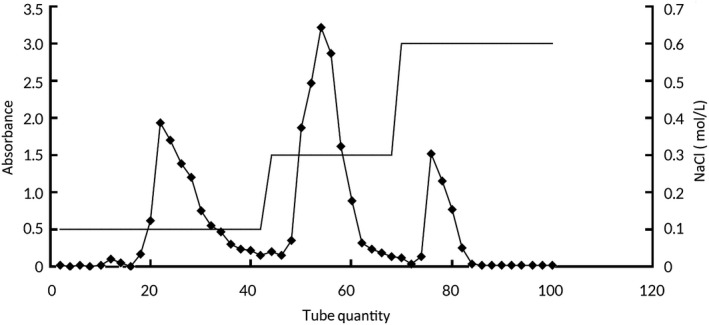
The purified result of AVP by DEAE‐52 fiber column chromatography

As shown in Figure 3, there are three acidic polysaccharide components in purified AVCP (AVP). They were named AVP‐1, AVP‐2, and AVP‐3 with percentages of 25.3%, 39.7%, and 15.8%, respectively.

The monosaccharide composition of AVP was qualitatively determined by GC (Table 3). AVP‐1 is mainly composed of L‐rhamnose, D‐xylose, D‐mannose, D‐glucose, D‐galactose, glucuronic acid, and fucose. AVP‐2 is mainly composed of four monosaccharides: L‐rhamnose, D‐glucose, glucuronic acid, and fucose. AVP‐3 is mainly composed of five monosaccharides, including L‐rhamnose, D‐glucose, D‐mannose, D‐galactose, and glucuronic acid.

**TABLE 3:** The results of monosaccharide composition

Components	Appearance time (min)			
	Standard monosaccharide	AVP‐1	AVP‐2	AVP‐3
L‐rhamnose	9.672	9.603	9.594	9.585
D‐mannose	11.479	11.365	—	—
D‐xylose	15.663	15.562	—	15.617
D‐glucose	16.452	16.374	16.345	16.351
D‐galactose	17.055	16.978	—	16.861
Fructose	23.480	—	—	—
Fucose	18.453	18.336	18.356	—
Glucuronic acid	14.231	14.112	14.129	14.116
Internal standard	28.750	28.673	28.652	28.669

### 3.3 IR analysis of AVP

**FIGURE 4:**
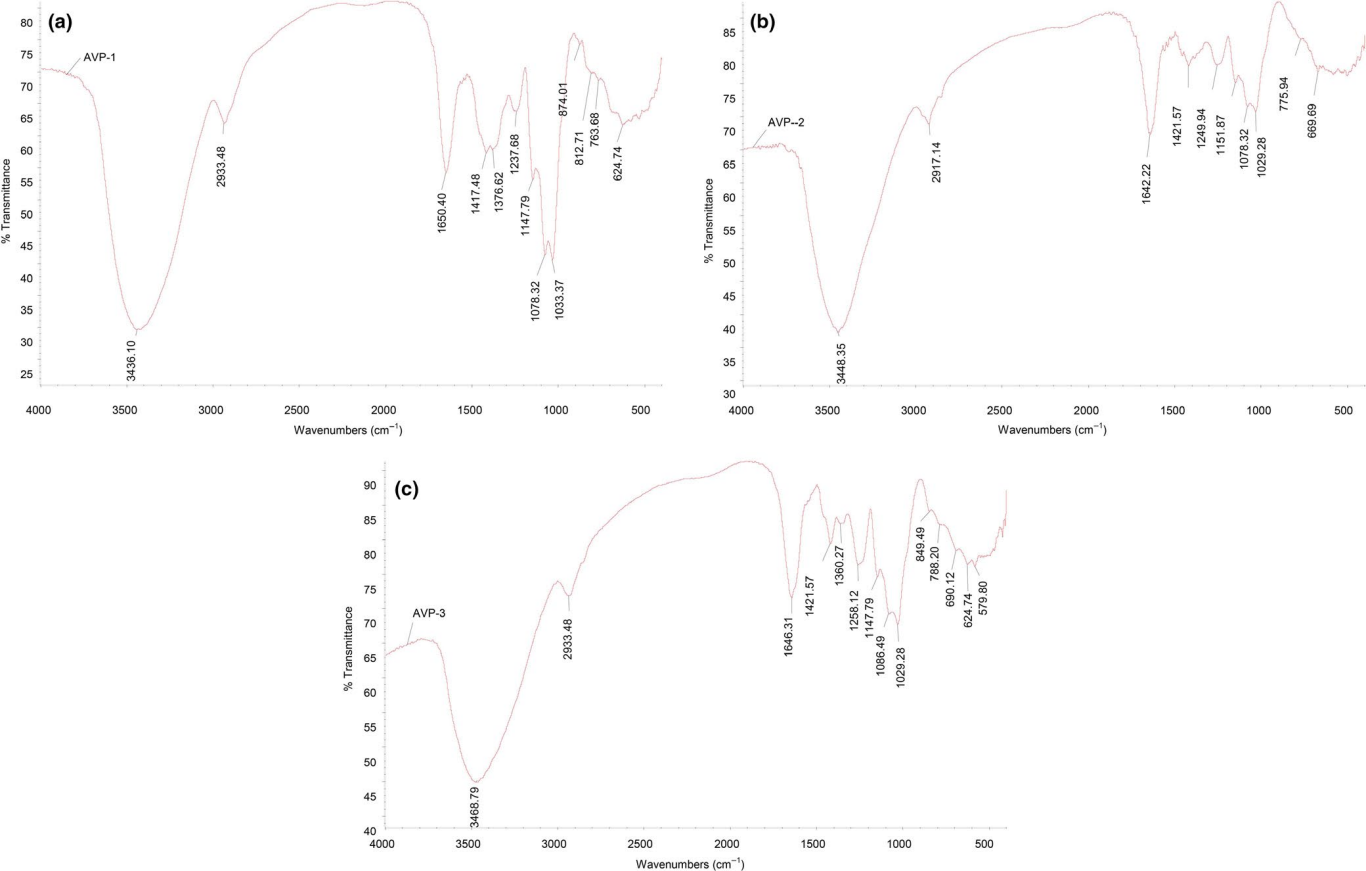
Infrared absorption spectrum of AVP‐1 (A), AVP‐2 (B), AVP‐3 (C)

The band characteristics of AVP‐1, AVP‐2 and AVP‐3 are illustrated in Table 4 and Figure 4A–C. It can be seen that all of AVP have O‐H, C‐H, ‐CHO, =CH_2_ bonds and pyran ring ether bond with C‐O‐C stretching vibration and O‐H variable angle vibration in their structure. It was also suggested that there was S=O stretching at 1,237.68 cm, 1,249.94 cm, and 1,258.12 cm^−1^ in AVP‐1, AVP‐2, and AVP‐3, indicating that the structures of the three AVP contained sulfate ion. The absorption band corresponding at 874.01 cm^−1^ represented absorption bands of mannopyranose and galactopyranoside, while the absorption bands at 812.71 and 763.68 cm^−1^ are the characteristic bands of mannan pyranose and α‐D‐xylulose, respectively, suggesting that AVP‐1 contained the above components. The weak absorption band at 849.49 cm^−1^ in AVP‐3 was assigned to the stretching vibration of the α‐type glycosidic bond. These results indicated that AVP possessed typical absorption peak of polysaccharides.

**TABLE 4:** IR analysis of AVP

Wavelength (cm^−1^)			Characterization of functional groups in AVP
AVP‐1	AVP‐2	AVP‐3	
3,436.53	3,448.35	3,468.79	O‐H stretching vibration
2,933.48	2,917.14	2,933.48	C‐H telescopic vibration
1,650.40	1,642.22	1,646.31	C=O asymmetric stretching vibration absorption peak of ‐CHO
1,417.48, 1,376.62	1,421.57	1,421.57	=CH_2_ deformation absorption peak of polysaccharides
—	—	1,360.27	C‐H bending vibration
1,237.68	1,249.94	1,258.12	S=O telescopic vibration
—	1,151.87	1,147.79	Absorption peak of C‐O on the ring
1,078.32, 1,033.37	1,078.32, 1,029.28	1,086.49, 1,029.28	C‐O‐C stretching vibration and O‐H variable angle Vibration of pyran ring ether bond
874.01	—	—	Absorption peaks of mannopyranose and galactopyranoside
—	—	849.49	Absorption peaks of α‐type glycosidic bonds
812.71	—	—	Absorption peaks of mannan pyranose
763.68	—	—	α‐D‐xylulose characteristic absorption

### 3.4 Hypolipidemic and anti‐atherogenic activities of AVCP

#### 3.4.1 Effect of AVCP on body weight of animals

**FIGURE 5:**
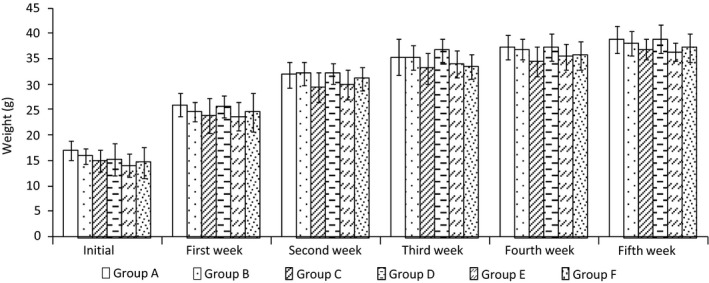
The effect of AVCP on the weight of mice during 5 successive weeks

After 5 weeks, the weights of mice in each group are shown in Figure 5, and it indicated that the various treatment interventions demonstrated no significant difference in the body weight of mice.

#### 3.4.2 Effect of AVCP on blood lipid profile

##### Effect of AVCP on the level of TC

After 5 weeks fed, the results in Table 5 demonstrated that compared to group C which was treated with high‐fat diet and 0.9% saline, the mice in groups A, B, D, E, and F showed significant decreases (*p* < .01) in TC levels (30.21%, 26.81%, 25.96%, 28.94%, and 29.79%). There were no significant differences between groups A, B, D, E, and F (*p *> .05), indicating that the efficacy of AVCP on reducing TC level was similar to hypotensive (Xuezhikang capsule).

**TABLE 5:** Effect of AVCP on TG, TC, HDL‐C, and LDL‐C level in rat serum

Groups	TG/(mmol/L)	TC/(mmol/L)	HDL‐C/(mmol/L)	LDL‐C/(mmol/L)
Group A	14.02 ± 0.91^e^	1.64 ± 0.09^e^	1.30 ± 0.17^f^	0.24 ± 0.07^e^
Group B	13.12 ± 0.64^e^	1.72 ± 0.09^e^	1.28 ± 0.15^f^	0.33 ± 0.03^e^
Group C	18.37 ± 1.71^ac^	2.35 ± 0.10^ac^	1.13 ± 0.14^bd^	0.43 ± 0.06^ac^
Group D	15.78 ± 2.35^f^	1.74 ± 0.12^e^	1.42 ± 0.09^e^	0.27 ± 0.05^e^
Group E	15.74 ± 2.40^f^	1.67 ± 0.10^e^	1.35 ± 0.09^e^	0.24 ± 0.05^e^
Group F	14.44 ± 0.87^e^	1.65 ± 0.06^e^	1.31 ± 0.09^e^	0.26 ± 0.05^e^

Note

Group A: blank control group; group B: positive control group; group C: negative control group; group D: low‐dose group; group E: middle‐dose group; group F: high‐dose group. a, *p* < 0.01, b, *p* < .05, compared with Group A; c, *p* < .01, d, *p* < .05, compared with Group B; e, *p* < .01, f, *p* < .05, compared with Group C.

##### Effect of AVCP on TG

The levels of TG are shown in Table 5. In comparison with group C, Xuezhikang capsule and AVCP treatment caused significant reductions on plasma TG levels (*p* < .05). The TG levels in groups D, E, and F (15.78 ± 2.35, 15.74 ± 2.40, and 14.44 ± 0.87 mmol/L) were close to that in group A (14.02 ± 0.91 mmol/L). As shown in Table 5, with increasing dose of AVCP, the level of TG was found to be correspondingly decreased. The results indicated that AVCP played a role in reducing TG level, relating to AVCP dose.

##### Effect of AVCP on the level of HDL‐C

Xuezhikang capsule in group B caused a significant increment (*p* < .05) in HDL‐C level compared to group C. As compared with group C, the same trends were obtained in groups D‐F with AVCP doses of 200, 400, and 600 mg/kg, indicating that AVCP had a significant effect on increasing HDL‐C level (*p* < .01; Table 5). With the dose of 200 mg/kg of AVCP, a significant rise in the level of HDL‐C (20.42%) was noted when compared with group C (*p* < .01), which was higher than that with 400 and 600 mg/kg of AVCP (19.47% and 15.93%), suggesting that there was an inverse correlation between the dose of AVCP and the level of HDL‐C.

##### Effect of AVCP on LDL‐C

As shown in Table 5, the LDL‐C level in group C (0.43 ± 0.06 mmol/L) was dramatically higher than that in the other groups (*p* < .01), and there were no significant differences of LDL‐C level in groups A, B, D, E, and F (0.24 ± 0.07, 0.33 ± 0.03, 0.27 ± 0.05, 0.24 ± 0.05, and 0.26 ± 0.05 mmol/L; *p *> .05). The results suggested that AVCP had a similar effect with Xuezhikang capsule on lowering the LDL‐C level of hypercholesterolemic mice.

#### 3.4.3 Effect of AVCP on lipid peroxidation

##### Effect of AVCP on the concentration of MDA

As shown in Table 6, compared to group C, the concentrations of MDA were significantly (*p* < .01) decreased by 17.36%, 25.67%, and 36.92% in the mice treated with AVCP at the doses of 200, 400, and 600 mg/kg (group D, group E, and group F), respectively. However, the levels of MDA in groups D and E were higher than the control group, group B, and group F, suggesting that the dose of AVCP ≥ 400 mg/kg would reduce the concentration of MDA to a normal level.

**TABLE 6:** Effect of AVCP on MDA, SOD in serum

Groups	MDA/(nmol/L)	SOD/(U/ml)
Group A	2.56 ± 0.16^e^	91.18 ± 5.09^e^
Group B	2.68 ± 0.14^e^	92.85 ± 6.49^e^
Group C	4.09 ± 0.23^ac^	63.51 ± 4.81^ac^
Group D	3.38 ± 0.22^ace^	86.42 ± 5.87^e^
Group E	3.04 ± 0.14^e^	82.68 ± 5.42^e^
Group F	2.58 ± 0.16^e^	85.98 ± 5.62^bde^

Note

a, *p* < .01, b, *p* < .05, compared with group A; c, *p* < .01, d, *p* < .05, compared with group B; e, *p* < .01, f, *p* < .05, compared with group C.

##### Effect of AVCP on SOD activity

Hyperlipidemic diets (group C) significantly decreased the activity of antioxidant enzyme SOD compared to normal diets (group A; *p* < .01). The data in Table 6 indicated the serum SOD activities were dramatically increased to 92.85 ± 6.49 U/ml in group B and 91.18 ± 5.09 U/ml in group A, which were higher than that in group C (63.51 ± 4.81 U/ml). Meanwhile, SOD activities were significantly reduced (*p* < .01) with the escalating dose from 200 to 600 mg/kg of AVCP (groups D–F). The SOD activities in groups D, E, and F were lower than that in groups A and B, but higher than that in group C, indicating that AVCP were less effective than Xuezhikang capsule in improving SOD activity. The results showed that as the dose of AVCP increased, the activity of SOD was decreased, suggesting the dose‐response relationship between AVCP and the activity of SOD.

### 3.5 Histopathological assessment

**FIGURE 6:**
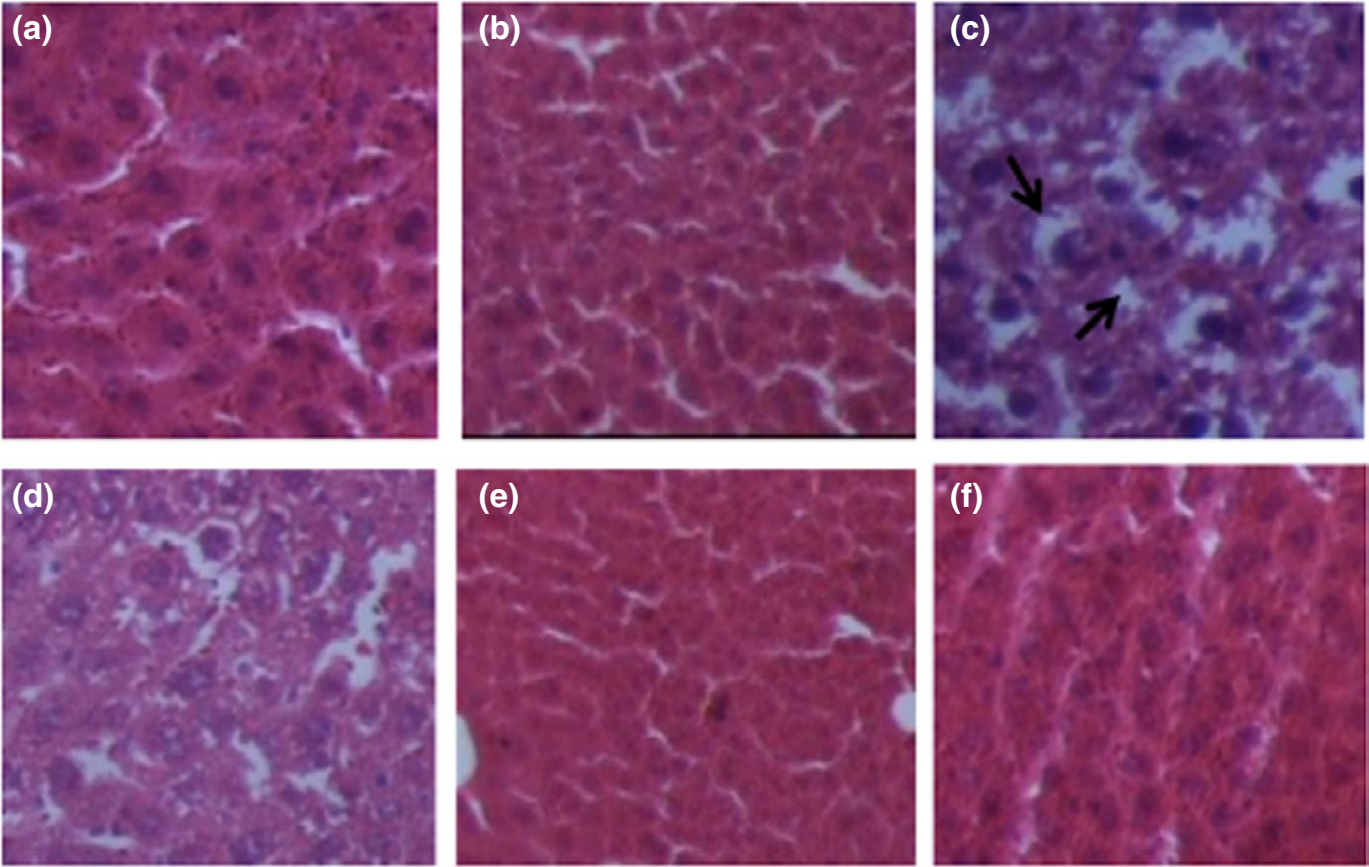
Photomicrograph of liver tissue of mice of group A, group B, group C, group D, group E, and group F

Microscopic images of liver tissues are shown in Figure 6A–F. Liver histological examination of group A showed normal hepatic cells and architecture, clear boundary, and well‐preserved cytoplasm with central nucleus (Figure 6A). However, group C showed the disordered liver structure with hepatocellular necrosis and extensive vacuolization which are indicated with the black arrow in Figure 6C. Xuezhikang capsule significantly attenuated lipid vacuolization (Figure 6B). With the treatments of 400 and 600 mg/kg AVCP, the disordered liver tissues returned to normalcy (Figure 6E,F). However, with low dose of AVCP in group D (Figure 6D), liver cell architecture was better than that in negative group C but worse than that in other groups. These results demonstrated that AVCP could reduce the accumulation of lipid droplets in hepatic tissue cells of hyperlipidemia mice and prevent cardiovascular disease. Moreover, the extent of liver tissue recovery was correlated with the dose of AVCP.

## 4 DISCUSSION

In this study, AVCP could be extracted by combination of PEF and neutral protease. The content of crude polysaccharides was around 60%. The results of GC analysis showed that the polysaccharides contained L‐rhamnose, D‐xylose, D‐mannose, D‐glucose, D‐galactose, glucuronic acid, and fucose. According to IR analysis, the absorption bands of O‐H, C‐H, ‐CHO, =CH_2_, S=O and pyran ring ether bonds were observed, indicating that the AVCP possessed typical absorption peak of polysaccharides.

Atherosclerosis is the main contributor to the pathogenesis of myocardial and cerebral infarction and highly related to blood lipids. Hyperlipidemia may be responsible for liver damage. In animal models of the high‐fat diet, a dramatic increase in serum low‐density lipoprotein (Morishita et al., 1986), triglyceride, and total cholesterol levels was reported with relatively slight changes in high‐density lipoprotein. Then, elevated levels of plasma TC and LDL‐C, accompanied by reducing TG and HDL levels, are often associated with an increased risk of atherosclerosis (Chander, Kapoorn, & Singh, 1988; Wu, 2007). And at the present time, most commonly used lipid regulators are the statin (simvastatin) and gemfibrozil (fibrates). However, recent reports of undesirable side effects (myopathy) of some “super‐statins” and drug‐induced liver and kidney damage (Tang, Gao, Wang, Wen, & Qin, 2013; Woo, Bok, & Choi, 2009). Therefore, it is necessary to search for a new value or functional bioactive compounds that could be used to amend this metabolic disorder without any side effect.

Numerous studies have shown that diet with high‐fat content caused a considerable increase in the serum TC, TG, and LDL‐C and a decrease in HDL‐C (Ban, Rico, Um, & Kang, 2012; Chawda, Mandavia, Parmar, Baxi, & Tripathi, 2014; Zhang, Zhang, Jiang, & Xia, 2013). In the present study, feeding mice with high‐fat diet resulted in upregulation of TC, TG, and LDL‐C and down‐regulation of HDL‐C level. In animal studies, it has been reported that some polysaccharides inhibit low‐density lipoprotein oxidation and have an overall positive effect on lipid metabolism and cholesterol (Liu et al., 2012). In the present study, we found that daily oral administration of different doses of AVCP inhibited serum TC, TG, and LDL‐C accumulation after 5 weeks and up‐regulated the level of HDL‐C, indicating that AVCP might be beneficial for degenerative diseases, as well as atherosclerosis.

Oxidative stress, the disturbance of the delicate balance between oxidants and antioxidants, could impair the antioxidant defense systems (Li et al., 2010). It was generally caused by the increasing levels of free radicals. Current studies have reported that the development of atherosclerosis was related to oxidative stress in plasma (Lusis, 2000). The major antioxidant enzymes, including SOD, GSH‐Px, and CAT, used as biomarkers to indicate free radical species (ROS) production, are regarded as the primary defense system against ROS generated in vivo during oxidative stress (Akindele, Otuguor, Singh, Ota, & Benebo, 2015; Tang et al., 2013). SOD is the only enzyme that disrupts oxygen free radicals and exists in all cells with high amounts in erythrocytes. Higher the SOD activity, faster the free radicals were scavenged (Fan et al., 2016). Previous studies in vivo indicated that feeding the animal with a high‐fat diet could lead to an increase of free radical production (Dobrian, Davies, Prewitt, & Lauterio, 2000; Feng, Yu, Ying, Hua, & Dai, 2011; Rony, Ajith, Nima, & Janardhanan, 2014) and a decrease of SOD activity (Bagchi, Bagchi, Hassoun, & Stohs, 1995), followed by hypercholesterolemia and oxidative stress (Ohkawa, Ohishi, & Yagi, 1979). Malondialdehyde (MDA), one of the end products of tissue lipid peroxidation, is regarded as a marker of lipid peroxidation and an index of the level of oxygen free radicals as well. Lower MDA level suggests less lipid peroxidation and weaker oxidant stress (Padmavathi, Senthilnathan, Chodon, & Sakthisekaran, 2006), followed by the reduction of atherosclerosis (Illingworth, 1993). The obtained results in this study indicated that high‐fat diet caused a marked reduction in serum SOD activity and an obvious increase in the concentration of MDA, suggesting a detrimental effect of intrinsic antioxidant defense system and endothelial cell injuries in mice. After 5 weeks of treatment of AVCP and Xuezhikang capsule (cholesterol‐lowering drug), the activities of SOD were significantly increased, and the levels of MDA were dramatically reduced. AVCP could preserve the activity of the antioxidant enzyme in high‐lipid organs, rectify the metabolic disturbance of free radicals, and maintain the dynamic balance of the oxidation and antioxidative systems.

## 5 CONCLUSION

This study demonstrated the potential lipid‐lowering effects of AVCP. By intake of AVCP, serum lipids of TG, TC, and LDL‐C and HDL‐C level was controlled, and SOD activity and the level of MDA were adjusted. The results implied that further study should be conducted to identify the possible mechanism and the therapeutic effect in the treatment of hyperlipidemia‐related diseases, such as atherosclerosis.

## CONFLICT OF INTEREST

Authors declare that they have no conflict of interest.

## ETHICAL APPROVAL

This study involving animal testings was approved by the Institutional Review Board of Fujian Medical University.
